# Data on the sensory evaluation of potatoes (*Solanum tuberosum*) from different areas of Hokkaido, Japan, performed by untrained young adults

**DOI:** 10.1016/j.dib.2017.09.047

**Published:** 2017-09-23

**Authors:** Hiroaki Sato, Ryosuke Koizumi, Yozo Nakazawa, Masao Yamazaki, Ryuichi Itoyama, Megumi Ichisawa, Junko Negishi, Rui Sakuma, Tadasu Furusho, Yoshimasa Sagane, Katsumi Takano

**Affiliations:** a Department of Food and Cosmetic Science, Faculty of Bioindustry, Tokyo University of Agriculture, 196 Yasaka, Abashiri Hokkaido 099-2493, Japan; b Research Department, Research & Development Division, Calbee, Inc., 23-6 Kiyohara Kougyoudanchi, Utsunomiya, Tochigi 321-3231, Japan; c Department of Nutrition, Junior College of Tokyo University of Agriculture, 1-1-1 Sakuragaoka, Setagaya-ku 156-8502, Tokyo, Japan; d Department of Applied Biology and Chemistry, Faculty of Applied Bioscience, Tokyo University of Agriculture, 1-1-1 Sakuragaoka, Setagaya-ku, Tokyo 156-8502, Japan

**Keywords:** Potato, Sensory evaluation, Tastes, Cultivars

## Abstract

This data article describes a sensory evaluation of potatoes used in food processing from the Tokachi, Kamikawa, and Abashiri geographic areas of Hokkaido, Japan, performed by untrained young adults. We gathered sensory data on potatoes from the four cultivars ‘Toyoshiro,’ ‘Kitahime,’ ‘Snowden,’ and ‘Poroshiri.’ The sensory evaluation was performed on steamed potatoes from each cultivar; these potatoes were harvested from each of the three geographic areas. Table 1 provides the data from the evaluation of the five basic tastes (sweet, salty, sour, bitter, and umami), as well as the evaluation of the *egumi* taste, which is a Japanese term indicating a taste that is acrid, astringent, and slightly bitter.

**Specifications Table**

**Table:** 

Subject area	Agricultural science
More specific subject area	Food science
Type of data	Table
How data were acquired	The taste (sweet, salty, sour, bitter, umami, and the *egumi* taste) of steamed potatoes was evaluated by 13 untrained individuals in a sensory test.
Data format	Analyzed
Experimental factors	Potato tubers were harvested in the Tokachi, Kamikawa, and Abashiri areas of Hokkaido, Japan during the autumn of 2014.
Experimental features	Sensory evaluation of the five basic tastes, and of the *egumi* taste, of potato tubers
Data source location	Tokachi, Kamikawa, and Abashiri areas of Hokkaido, Japan
Data accessibility	The data are presented in this article.

**Value of the data**

• The data presented will be available as reference to compare the tastes of different potato cultivars.
• The data presented may serve as an index for selecting potato cultivars for producing a desired taste.
• The data presented are available for use in food processing when selecting potatoes based on the taste of each cultivar and production area.

## 1 Data

This article presents data on the sensory evaluation of potato tubers from four cultivars (‘Toyoshiro,’ ‘Kitahime,’ ‘Snowden,’ and ‘Poroshiri’) by untrained young adults (Table 1). Potatoes from each cultivar were harvested at farms in the Tokachi, Kamikawa, and Abashiri areas of Hokkaido, Japan. Japanese terms in this article are explained in Table S1.

**Table 1:** Sensory evaluation of potato tubers by untrained young adults.

Parameter	Cultivation area	Cultivar			
		Toyoshiro	Kitahime	Snowden	Poroshiri
Sweetness	Tokachi	2.08±0.76^A, a^	1.85±0.55^A, ab^	1.77±0.60^A, ab^	1.31±0.48^A, b^
	Kamikawa	2.00±0.91^A, a^	1.54±0.52^AB, a^	1.92±0.76^A, a^	n.d.
	Abashiri	1.77±0.60^A, a^	1.23±0.44^B, a^	1.62±0.65^A, a^	1.23±0.44^A, a^
Saltiness	Tokachi	1.46±0.52^A, a^	1.23±0.44^A, a^	1.54±0.66^A, a^	1.15±0.38^A, a^
	Kamikawa	1.38±0.65^A, a^	1.15±0.38^A, a^	1.54±0.52^A, a^	n.d.
	Abashiri	1.54±0.66^A, a^	1.15±0.38^A, a^	1.23±0.60^A, a^	1.15±0.38^A, a^
Sourness	Tokachi	1.23±0.60^A, a^	1.00±0.00 ^B, a^	1.15±0.38^A, a^	1.54±0.78^A, a^
	Kamikawa	1.38±0.65^A, a^	1.31±0.63 ^B, a^	1.00±0.00^A, a^	n.d.
	Abashiri	1.38±0.77^A, b^	2.08±0.76^A, a^	1.23±0.60^A, b^	1.08±0.28^A, b^
Bitterness	Tokachi	1.23±0.44^A, b^	1.62±0.77^A, ab^	1.38±0.51^A, ab^	1.85±0.55^A, a^
	Kamikawa	1.46±0.66^A, a^	1.69±0.75^A, a^	1.85±0.69^A, a^	n.d.
	Abashiri	1.62±0.65^A, a^	2.15±0.69^A, a^	1.69±0.75^A, a^	1.85±0.99^A, a^
Umami	Tokachi	2.08±0.64^A, a^	1.92±0.64^A, a^	1.85±0.55^A, a^	1.08±0.28 ^B, b^
	Kamikawa	1.85±0.80^A, a^	1.46±0.52 ^AB, a^	1.92±0.86^A, a^	n.d.
	Abashiri	1.77±0.60^A, a^	1.08±0.28 ^B, b^	1.54±0.52^A, ab^	1.46±0.52^A, ab^
*Egumi* Taste^a^	Tokachi	1.31±0.48^A, a^	1.62±0.77 ^B, a^	1.92±0.86^A, a^	2.00±1.00^A, a^
	Kamikawa	1.69±0.75^A, a^	1.69±0.75 ^B, a^	2.00±0.71^A, a^	n.d.
	Abashiri	1.85±0.80^A, ab^	2.46±0.66^A, a^	1.69±0.63^A, b^	1.92±0.76^A, ab^

The different upper-case letters indicate significant differences (*P* < 0.05) between cultivation areas, for each cultivar and specific taste.

The different lower-case letters indicate significant differences (*P* < 0.05) in the same row.

n.d.: No data

a The *egumi* taste is an acrid, astringent, and bitter-like taste that irritates the root of tongue [2], [3]

## 2 Experimental design

We selected the four cultivars that are most widely farmed for food-processing purposes. In Japan, ‘Toyoshiro’ is the most popular cultivar and provides the highest yield for processing purposes [1]. ‘Kitahime,’ ‘Snowden,’ and ‘Poroshiri’ are predominantly used for the production of potato chips. These four cultivars were sourced from the Tokachi, Kamikawa, and Abashiri regions. Our taste testers assessed the five basic tastes, i.e., sweet, salty, sour, bitter, and umami, as well as *egumi*, which is a Japanese term indicating an acrid and astringent taste that is slightly bitter [2], [3]. For the evaluation of these tastes, we employed a questionnaire.

## 3 Materials and methods

### 3.1 Materials

Potatoes were harvested from the ‘Toyoshiro,’ ‘Kitahime,’ ‘Snowden,’ and ‘Poroshiri’ cultivars, from farms in the Tokachi, Kamikawa, and Abashiri areas of Hokkaido, Japan, during the autumn of 2014.

### 3.2 Sensory evaluation

After harvest, tubers were preserved at −80 °C, until they were used in the sensory evaluation. Tubers with a specific gravity around the average value for each group were steamed for 40 min. The sensory evaluation was performed by a panel of thirteen untrained university students. The measured parameters were “sweetness,” “saltiness,” “sourness,” “bitterness,” “umami,” and the “*egumi* taste.” These taste parameters were judged using a three-point scale: 1, no taste; 2, slight taste; and 3, strong taste. Measurements were statistically analyzed by Tukey's test (*P*<0.05).
