# Supplementary material for: Data on the sensory evaluation of potatoes (Solanum tuberosum) from different areas of Hokkaido, Japan, performed by untrained young adults
Source: Data Brief. 2017 Sep 23;15:397–400. doi: 10.1016/j.dib.2017.09.047 (PMC5712046; doi:10.1016/j.dib.2017.09.047)
Supplement: Supplementary file 2 — Supplementary material [file mmc2.docx]

**Table S1. Glossary of Japanese terms in this article.**

| Japanese terms | Meanings |
| --- | --- |
| ‘Toyoshiro’ | A name of one of potato cultivars in Japan |
| ‘Kitahime’ | A name of one of potato cultivars in Japan |
| ‘Poroshiri’ | A name of one of potato cultivars in Japan |
| Umami | A taste sensation that is meaty or savory and in produced by several amino acids and nucleotides (such as glutamate and aspartate) |
| Egumi | A taste sensation that is acrid, astringent, and bitter-like taste that irritates the root of tongue |
